# LibME—automatic extraction of 3D ligand‐binding motifs for mechanistic analysis of protein–ligand recognition

**DOI:** 10.1002/2211-5463.12150

**Published:** 2016-11-30

**Authors:** Wei He, Zhi Liang, MaiKun Teng, LiWen Niu

**Affiliations:** ^1^ Hefei National Laboratory for Physical Sciences at Microscale and School of Life SciencesUniversity of Science and Technology of ChinaAnhuiChina

**Keywords:** algorithm, binding motif, protein–ligand recognition

## Abstract

Identifying conserved binding motifs is an efficient way to study protein–ligand recognition. Most 3D binding motifs only contain information from the protein side, and so motifs that combine information from both protein and ligand sides are desired. Here, we propose an algorithm called LibME (Ligand‐binding Motif Extractor), which automatically extracts 3D binding motifs composed of the target ligand and surrounding conserved residues. We show that the motifs extracted by LibME for ATP and its analogs are highly similar to well‐known motifs reported by previous studies. The superiority of our method to handle flexible ligands was also demonstrated using isocitric acid as an example. Finally, we show that these motifs, together with their visual exhibition, permit better investigating and understanding of protein–ligand recognition process.

**Abbreviations**
ICT: isocitric acid
LAC: lactic acid
PDB: protein data bank
PHE: phenylalanine
TCA: tricarboxylicacid
TYR: tyrosine

Protein–ligand recognition plays vital roles in many biological processes in living cells including enzyme catalysis, signal transduction, molecular transportation, and so on. Identifying conserved ligand‐binding motifs that are reused across protein pockets binding the same or similar ligands is critical for understanding molecular recognition mechanisms. Many methods have been developed to extract 3D ligand‐binding motifs by comparative analysis of protein pockets 1, 2, 3, 4, 5, 6. The 3D binding motifs extracted through these methods were proven to be efficient for pockets identification, comparison, and classification, thus applied to binding ligand prediction for a given pocket. However, these binding motifs only contain information from protein side, so they cannot provide details about interactions between ligand and its receptor, which is important for understanding protein–ligand recognition. For a given ligand, binding motifs composed of conserved residues surrounding the ligand had been proven to be reused in many globally diverse proteins, for example, comparative analysis showed that the residues of phosphate‐binding loop (P‐loop), a well‐studied functional motif in diverse phosphate‐binding proteins are highly conserved in terms of amino acid type 7, 8. When looking from the view of the ligand, the spatial positions of these residues are almost constant relative to the phosphate group, which provides further information for investigating the phosphate‐binding mechanism. However, discovery of this kind of 3D motifs mainly depends on manual analysis, which imposes restriction on large‐scale extraction of such motifs. So the question is: how can we automatically extract the motif integrating information of the ligand and the conserved residues surrounding it from a set of globally diverse proteins binding the same or a similar ligand?

One natural solution is ligand‐induced superimposing of proteins that bind the same ligand followed by clustering of conserved residues or atoms interacting with the ligand. Using this strategy, Kuttner and colleagues derived a set of atom clusters characterizing the adenine‐binding pockets by superimposing protein‐ATP complexes with the adenine moiety as a template and then extracting clustered binding‐site atoms of compatible atomic classes forming attractive contacts with the ligand 9. Nebel *et al*. 10 developed a similar method that could automatically extract 3D binding motifs from a set of protein–ligand complexes, which first aligns the proteins under the guidance of the common ligand, then clusters pocket atoms interacting with the ligand according to their chemical types and spatial positions, and finally generates consensus ligand‐binding patterns by assembling equivalent pocket atom clusters. However, the strategy works well only for ligands with rigid structures, so that the quality of structural alignment can be guaranteed. Unfortunately, only a small number of ligands are rigid. Although some structural alignment algorithms are tolerable to structural flexibility to some extent, the errors introduced in the stage of structural alignment might generate unpredictable influences on the subsequent analysis.

Here, we introduce a method called LibME (Ligand‐binding Motif Extractor) to extract 3D ligand‐binding motifs which combines information from both the pocket and the ligand sides by encoding the chemical types and the positions of pocket residues relative to the ligand, respectively. By incorporating the ‘relative position to the ligand’, we avoid the ligand‐induced alignment of the pockets thus conquering the shortness of methods mentioned above. A motif identified by LibME is composed of the ligand and the conserved residues surrounding it, which provides details of protein–ligand interactions. In this work, we first demonstrate the feasibility of our method by showing that the conserved 3D binding motifs for ATP and its analogs extracted by LibME are consistent with those well‐validated functional motifs obtained through manual analysis. Then, we show the advantage of our method in handling flexible ligands by extracting motifs for isocitric acid (ICT), a ligand without a rigid part. Finally, we illustrated that the motifs extracted by our method permit better investigating and understanding of protein–ligand recognition.

## Materials and methods

### Description of the algorithm

**Figure 1:**
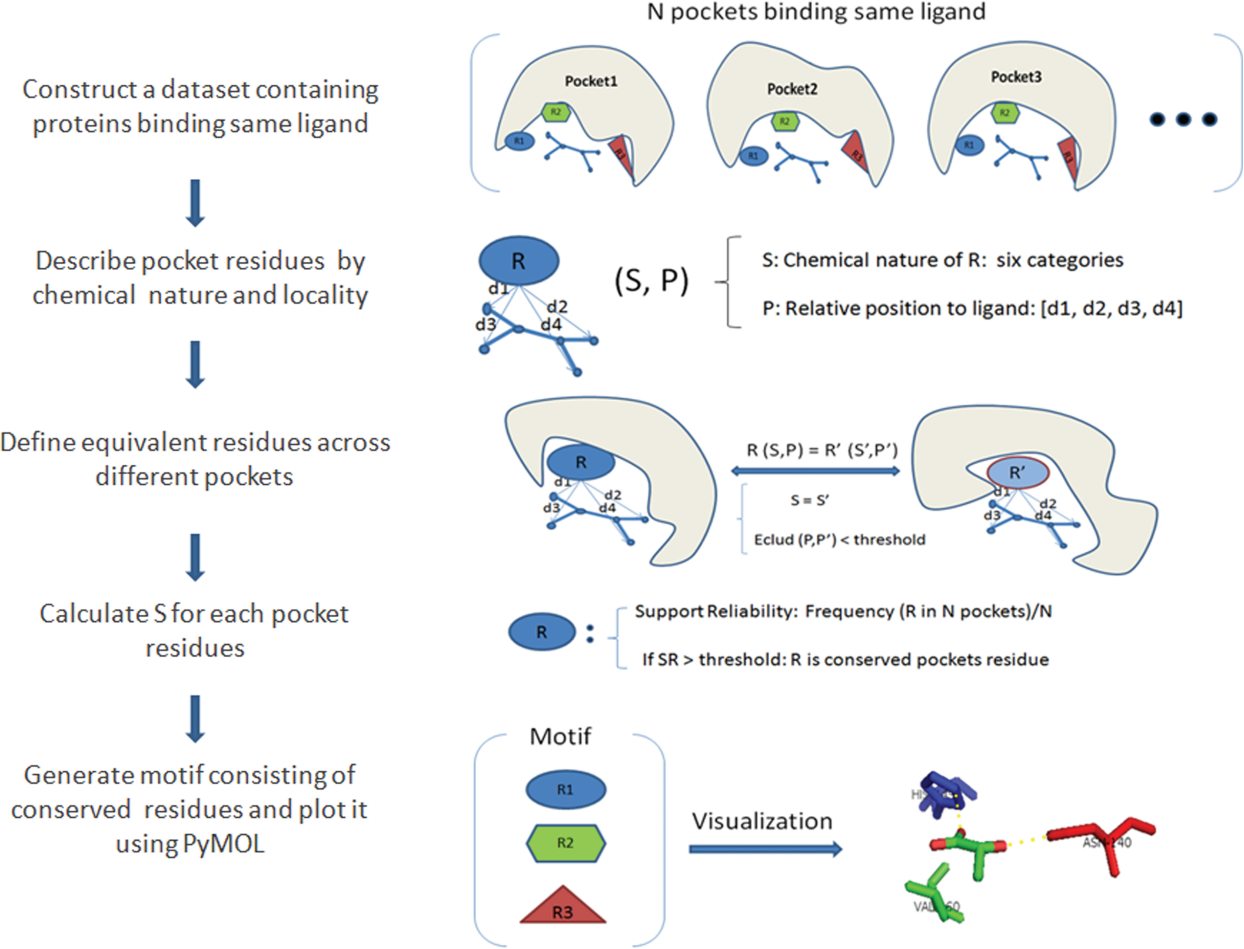
Workflow of LibME algorithm.

The molecular function of a protein is often carried out through a limited number of amino acids, which are reused in functional conserved proteins during evolution 11. Based on this observation, the LibME algorithm tries to identify pocket residues that are situated around the target ligand which are conserved in terms of chemical property and spatial position. Since metal ions also play important roles in the binding of many ligands 12, we extend the definition of a residue so that a residue can be an amino acid or a metal ion. The workflow of LibME is shown in Fig. 1: Given a set of proteins binding the same ligand, the residues with at least one atom within 5 Å to any atom of the ligand are considered as pocket residues 2, 4. Two pocket residues from two different proteins are considered to be equivalent if they belong to the same chemical category and have similar spatial position relative to the ligand 13. The conservation of a pocket residue is evaluated as the fraction of proteins that harbor equivalent residues of this residue. Given a conservation level, the corresponding ligand‐binding motifs are then be extracted by assembling proper pocket residues. The detailed procedure is described as follows.

#### Description of a pocket residue

We describe a pocket residue ***R*** with a tuple (*S*,***P***) consisting of two elements, which capture the chemical property of the residue and its relative position to the ligand, respectively.

The first element *S* depicts the chemical category that the residue belongs to

*S* = *s*

where *s* ranges from 1 to 7. We classified the 20 amino acids into six categories according to the biochemical properties of their side chains: (a) acidic (D and E), (b) basic (R, H, and K), (c) amidic (N and Q), (d) hydroxyl (S, C, T, and Y), (e) aromatic (F, W, and Y), (f) hydrophobic (A, G, I, L, P, V, and M) 13. A seventh category is introduced for metal ions: (g) CA, FE, ZN, CU, MN, and MG.

The second element ***P*** describes the relative spatial position of a pocket residue to the ligand

$$P=[d_{1},d_{2},d_{3},d_{4}]$$

where *d*_*1*_, *d*_*2*_, *d*_*3*_, and *d*_*4*_ are the distances from the Cα of an amino acid or the metal ion to four noncoplanar atoms of the ligand, respectively. The ‘four‐atoms’ are selected as follows: First, all the ligand's atoms are sorted according to their spatial arranging sequences from left to right and then we select the first, the one‐third, the two‐third, and the last of sorted atoms so that four selected atoms will not be located on the same planar except for some extreme cases. The use of this four‐atom system to express the spatial position of a residue is based on the fact that the position of a point can be unambiguously determined by its distances to four known noncoplanar points.

#### Determination of equivalent residues

Based on the above representation of a pocket residue, the equivalence of two residues ***R***_***1***_ = (*S*_*1*_, ***P***_1_) and ***R***_***2***_ = (*S*_*2*_, ***P***_2_) are determined using the following function

$$\text{IsEquivalent}\;\left(R_{1},R_{2}\right)=\left(S_{1}=S_{2}\;\text{and D}\left(P_{1},P_{2}\right)<\sigma \right),$$

where D(***P***_1_, ***P***_2_) is the Euclidian distance between ***P***_1_ and ***P***_2_. Two residues are defined to be equivalent, if they have the same chemical property and are close enough in space with respect to the ligand. σ is the maximal tolerance allowed to regard two residues to be equivalent in terms of their spatial positions.

#### Support reliability of a pocket residue

Given a specific pocket residue, the support reliability or conservation of the residue is defined as

$$\text{SR}=\frac{F}{N}$$

where *N* is the number of proteins in the dataset and *F* represents the number of proteins that harbor the corresponding equivalent residues. This index can also be explained as the probability that one residue with certain chemical property appear in certain position around the target ligand.

#### Generation of 3D binding motifs under certain support reliability

To generate 3D binding motifs, a lower bound of support reliability SR should be chosen first, for every pocket residue in every protein in the dataset, the SR value is calculated as described above, residues with SR value higher or equal to the lower bound are kept. Then, the residues which are equivalent to each other are merged into one cluster so that all the kept residues can be classified into several clusters. In this study, we merged two subclusters into one cluster if there are more than one equivalent residues in each subcluster. For example, (R1, R2) and (R2, R3) are two independent clusters while (R1, R2, R3) and (R1, R2, R4) can be merged as (R1, R2, R3, R4). For each residue cluster, the residue with the highest SR value is selected as the representative, whose features can be described as a tuple (*S*, <***P***>, <SR>), three elements represent the chemical category, the spatial position to the ligand and the SR value, respectively. So the finally obtained 3D binding motif is composed of a series of representative residues with certain features. In our analysis, the default lower bound for SR is set to be 0.5 to guarantee that the conserved residue appears in at least half of proteins in the dataset.

#### Visualization of the 3D binding motifs

Since the generated motifs contain information about the chemical types of the residues as well as their positioning relative to the ligand, we can directly visualize the motifs using the information. A ligand with known coordinates is firstly displayed and fixed. Then, all the representative residues are moved into the same coordinate system of the ligand according to their ‘four distances’ to the four‐atom reference system in the ligand. So we can visualize the motif containing the target ligand and conserved residues located around it through PyMOL 14.

### Results assessment

In the present work, we selected ATP and ATP‐binding proteins as a model system, from which many previous work had been done to extract functional binding motifs 15, 16, 17, 18. We downloaded all the protein structures cocrystallized with ATP from Protein Data Bank (PDB) 19. These structures were then clustered with 30% sequence identity using Cd‐hit 20. To eliminate redundancy, only one representative was kept for each cluster. Finally, 50 nonredundant ATP‐binding proteins were obtained to construct the training dataset used for motif extraction, another 50 nonredundant structures binding ATP were randomly selected to construct the testing dataset used for validating the motifs extracted (Table S1).

We firstly extracted binding motifs from the training dataset as described in the method section, which consist of several representative residues with certain chemical nature and spatial position to the ligand as well as a corresponding SR value. For each representative residue in the motif, we tested whether an equivalent residue appears in the proteins in the testing dataset, the fraction of proteins harboring certain representative residue can be calculated subsequently. Given a motif with *N* representative residues, we got two vectors: the first one describes SR values for each residue: [SR_1,_SR_2,_SR_3,_…, SR_*N*_], the second one describes fractions of proteins in the testing dataset harboring corresponding residues: [FR_1,_FR_2_,FR_3_,…, FR_*N*_]. We then compare these two vectors by calculating the Tanimoto coefficient as following:

$$T_{c}=\frac{\sum_{i=1}^{N}{\text{SR}}_{i}{\text{FR}}_{i}}{\left(\sum_{i=1}^{N}{\left({\text{SR}}_{i}\right)}^{2}+\sum_{i=1}^{N}{\left({\text{FR}}_{i}\right)}^{2}-\sum_{i=1}^{n}{\text{SR}}_{i}{\text{FR}}_{i}\right)}$$

*T*_C_ is used to evaluate the consensus of binding motif among training set and testing set, the value of which is between 0 and 1. The higher value indicates more consensus and vice versa.

### Data accessibility

Source code and data used are freely available from http://staff.ustc.edu.cn/~liangzhi/libme/.

## Results and discussion

### Determination of the parameter

In this work, we introduced one important parameter σ, which defines the maximal tolerance allowed to regard two residues to be equivalent in terms of their spatial positions.

We set σ based on two estimates: the first one (RMSD_1_) takes into account random position variations of pocket residues. According to the study of Eyal *et al*. 21, the RMSD of Cαs between the same protein structures determined at least twice could reach 0.9 Å. So the lower bound for σ is twice that value as $\sqrt[{2}]{4}\left(\text{RMDS}1\right)$. The second one (RMSD_2_) considers the variations due to the four ligand atoms selected as the reference. We take the average RMSD by pairwise alignments of all the ‘four‐atom’ systems in the dataset as RMSD_2_. If we consider these two estimates together, the maximum allowable error for Euclidian distance between ***P***_1_ and ***P***_2_ could reach $\sqrt[{2}]{4(}\text{RMDS}1+\text{RMSD}2)$. So we select a series of values between $\sqrt[{2}]{4(}\text{RMDS}1)$ and $\sqrt[{2}]{4(}\text{RMDS}1+\text{RMSD}2)$ with a step of 0.25 Å. In the case of ATP, we selected 2.0, 2.25, 2.5, 2.75, 3.0, 3.25, and 3.5 Å, respectively.

Table 1 reports the important information about motifs extracted under different value of σ including the number of representative residues and the *T*_C_ value. The number of residues in the motif indicates the flexibility of the method, more number of residues means higher extent of flexibility. The *T*_C_ value indicates the accuracy of the method, higher value means higher accuracy. The optimal parameter should obtain moderate flexibility with high accuracy. As we can see from the table, when σ equals to 2.75 Å, we got moderate number of residues in motif and obtained relatively high *T*_C_ value, which makes good compromise between flexibility and accuracy.

**Table 1:** Information of the motif under different value of σ

σ value in Å	Number of residues in motif	*T*_C_ value
2.00	3	0.945
2.25	5	0.959
2.50	5	0.974
2.75	9	0.960
3.00	10	0.937
3.25	13	0.945
3.50	12	0.940

Another factor which may affect the results is the size of dataset, in order to evaluate how dataset size affects the results, we also tested the results in another five datasets DS1, DS2, DS3, DS4, DS5 with 5, 10, 20, 30, 40 nonredundant structures, respectively. As we can see in Table 2, the method got highly consistent performance in DS2, DS3, DS4, and DS5 with high *T*_C_ values (over 0.94). However, when the number of structures is < 10 (we selected five here), the consensus become worse with *T*_C_ value of 0.81. Even though some highly conserved motifs can still be obtained in this tiny dataset, it is suggested that abundant number of structures are required to guarantee the accuracy as well as comprehensiveness of the extracted motifs.

**Table 2:** *T*c value under different sizes of datasets

Dataset	Number of structures	*T*_C_ value
DS1	5	0.811
DS2	10	0.948
DS3	20	0.966
DS4	30	0.975
DS5	40	0.973

**Figure 2:**
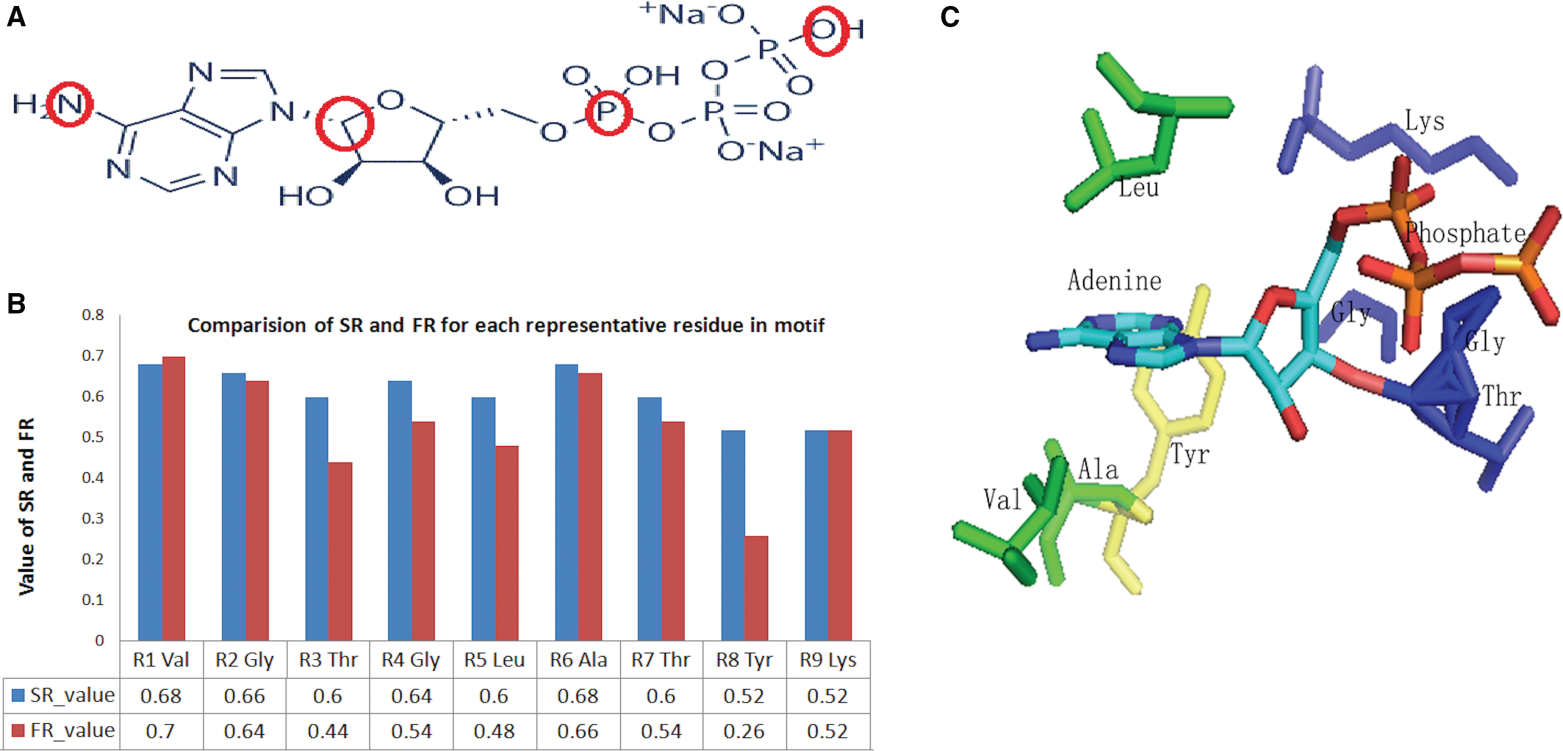
(A) ‘Four‐atoms’ system selected for ATP, the atoms circled red are selected as reference atoms. (B) Comparision of SR value and FR values for each representative residue in the binding motif for ATP. (C) The 3D binding motif extracted by LibME for ATP under SR = 0.5. ATP and the conserved residues surrounded are rendered as sticks by PyMOL. The hydrophobic, aromatic and phosphate‐binding submotfis are rendered in green, yellow, and red, respectively.

Besides, the ligand atoms selected as reference may also affect the results. In this study, we selected ‘four‐atoms’ system to express the relative position to the ligand for the following reasons: (a) the tolerance to the flexibility is closely related to the number of atoms involved in the system, more atoms result in a larger deviation since the distance to each atom of ligand contributes to the total deviation. So we selected the least number of atoms that determine the position of the residue. (b) It is hard to consider every possible ‘four‐atoms’ in a ligand especially when the size of the ligand is large, so we unified the selection of the first, one‐third, the two‐third, and the last of all the atoms which are sorted according to their spatial arranging sequences from left to right, this kind of selection guarantees that every part of ligand involved in the system which prevents ‘bias’ to some extent. In Fig. 2a, we displayed an example to how ‘four‐atoms’ selected for ATP.

### Analysis of extracted motif for ATP

We extracted the binding motif for ATP as described above, as we can see from the (a) in Fig. 2, nearly all the representative residues in the binding motif got consensus SR value and FR value except for R8, indicating the stability and conservativeness of the motif among two randomly selected datasets. The aromatic residue which R8 stands for seems less conserved than other residues. We can also view these representative residues surrounding ATP directly as shown in (b) in Fig. 2, three hydrophobic residues (LEU, VAL, ALA) located above and below adenine and interact with the adenine base through C‐H–π interactions between hydrophobic side‐chain groups and the face of the adenine ring. This submotif consisting of hydrophobic residues is consistent with the hydrophobic motif proposed by Moodie *et al*. and Denessiouk *et al.,* the former described the recognition of adenine by proteins in terms of a fuzzy recognition template based on a sandwich‐like structure formed by hydrophobic residues 15, the latter found that bulky hydrophobic residues can form a hydrophobic area by interacting with the adenine base 16. We can also see the conserved aromatic TYR residue under the adenine base, the submotif is in agreement with the A‐loop motif which is considered to play an important role in the binding of adenine through π–π interactions between aromatic rings and the adenine base 17, 18. Besides, four residues (GLY, LYS, THR, GLY) rendered in blue compose another submotif located around the phosphate group, among which Lys and Thr are also conserved residues in the P‐loop motif that typically consists of a glycine‐rich sequence followed by a conserved lysine and a serine or threonine 7, 8 In general, the motif identified at SR = 0.5 includes all the three previously validated motifs, indicating the capability of our method to extract biologically meaningful motifs for ligand binding.

In order to verify the significance of these conserved interactions involved in the ATP‐binding motif described above, we studied the effects of mutations on the conserved residues by conducting case studies referring to Platinum, a database of experimentally measured affinity change upon mutations on structurally resolved protein–ligand complexes 22. 1AMW is an ATP‐dependent molecular chaperone (HSP82). The ATP‐binding affinity (expressed in disassociation constant) of HSP82 decreases from 0.018 to 0.04 mm by I89V and L39I mutations, while V136M diminishes the affinity by almost 10 folds from 0.018 to 0.14 mm. Besides, mutation K98N also causes a decline in affinity from 0.018 to 0.05 mm. Among all these residues, I89, L39, and V136 are involved in the hydrophobic motif, while K98 is the conserved residue in P‐loop. Mutations of these residues reduce the affinity to a certain extent, indicating important roles they took in the ligand‐binding process. 3DGL is another ATP‐binding protein. One mutation on the aromatic residue beneath the adenine base from TYR to PHE causes an increase in the binding affinity from 0.25 to 0.18 mm. Although TYR and PHE are both aromatic residues, the slight difference affects the binding affinity showing that the aromatic residue is also important for ATP binding.

### Binding motifs for ligands similar to ATP

In the case of ATP, we obtain conserved 3D binding motif composed of submotifs that target specific functional groups. To test whether these motifs are identical for ligands with similar functional groups, we extract binding motifs for two ligands similar to ATP: one is AMP which also contains adenine base and another one is GTP which harbors a phosphate group like ATP.

**Figure 3:**
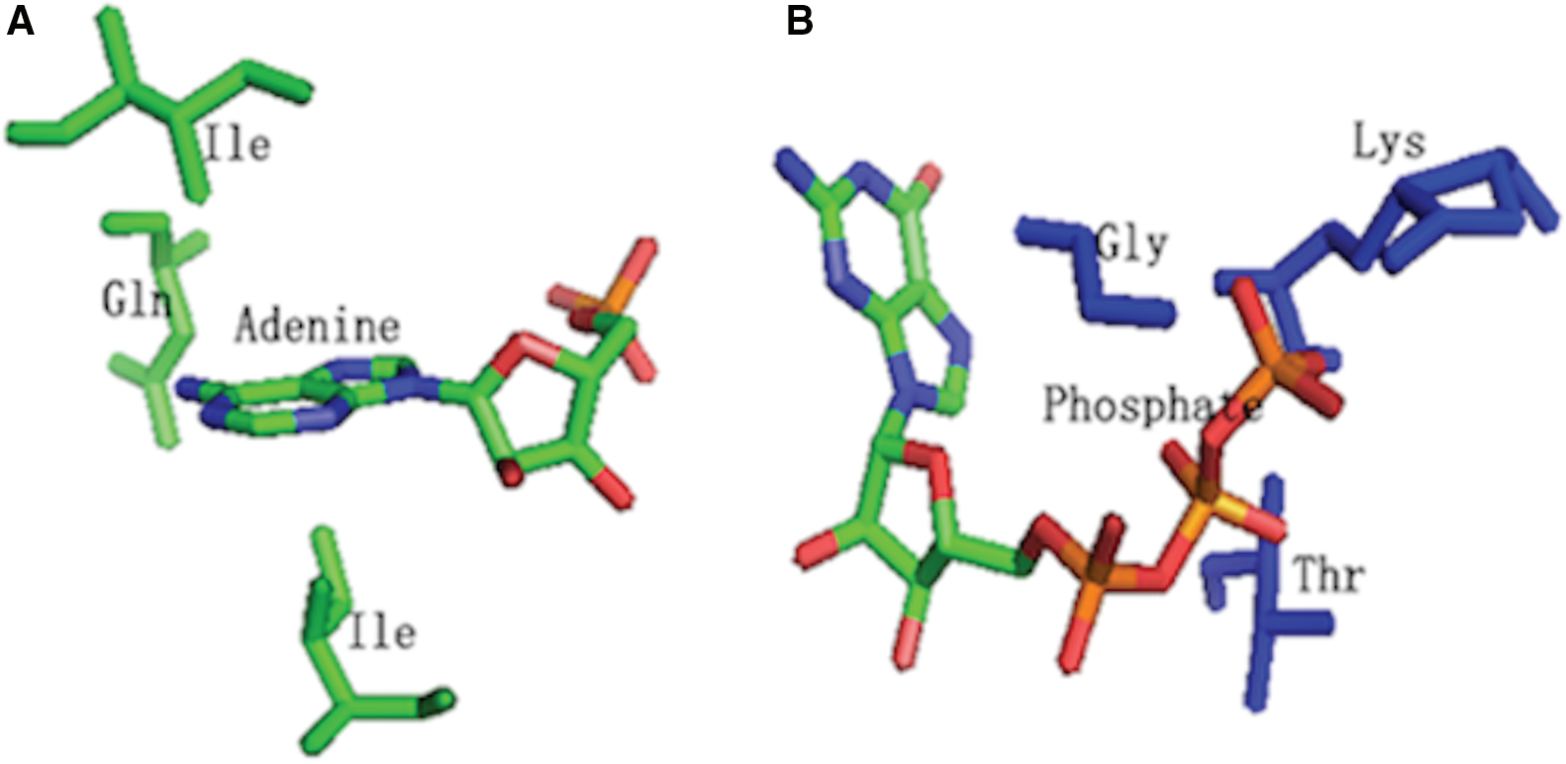
(A) The 3D binding submotif targeting adenine base extracted by LibME for AMP under SR = 0.5. (B) The 3D binding submotif targeting phosphate group extracted by LibME for GTP under SR = 0.5. The conserved residues surrounded are rendered as sticks by PyMOL. The hydrophobic submotif and phosphate‐binding submotfis are rendered in green and blue, respectively.

Using the same procedure described above, we obtained a dataset consisting of 48 and 19 nonredundant proteins cocrystallized with AMP and GTP, respectively (Table S1) and extracted binding motifs by LibME. As we can see in Fig. 3, (a) showed the submotif targeting adenine base for AMP and (b) plotted the submotif targeting phosphate group for GTP. In the submotif targeting adenine base for AMP, two conserved hydrophobic residues were located above and below the adenine base just as the situation for ATP, which is not beyond our expectation.

Besides, three conserved residues (LYS, SER, GLY) of a P‐loop motif also appear in the submotif‐targeting phosphate group for GTP as shown in (b). These results showed that the conserved binding motifs for specific functional groups are identical in general. Since enough number of protein–ligand complexes are often required to extract precise binding motifs for a specific ligand, it is possible using the algorithm with ligands containing abundant data to predict the motif for ligands with similar functional groups, thus greatly broadening the application range of our method.

### Binding motif extraction for ligands with flexible parts

As mentioned above, in comparison with ligand‐induced superimposition of proteins, LibME is superior in handling flexible ligands. In the case of ATP, the method already showed potential to extract motifs targeting flexible part (phosphate group). In this section, we applied LibME to extract biologically meaningful motifs interacting with flexbile ligands by taking ICT as an example, ICT is a flexible small molecule playing an important role in the TCA cycle. The number of ICT‐binding proteins deposited at PDB is less than that of ATP binding. Using the same procedure described above, we obtained a dataset consisting of 10 nonredundant proteins cocrystallized with ICT (Table S1) and extracted binding motif by LibME, the suitable value for σ is calculated as 2.25.

**Figure 4:**
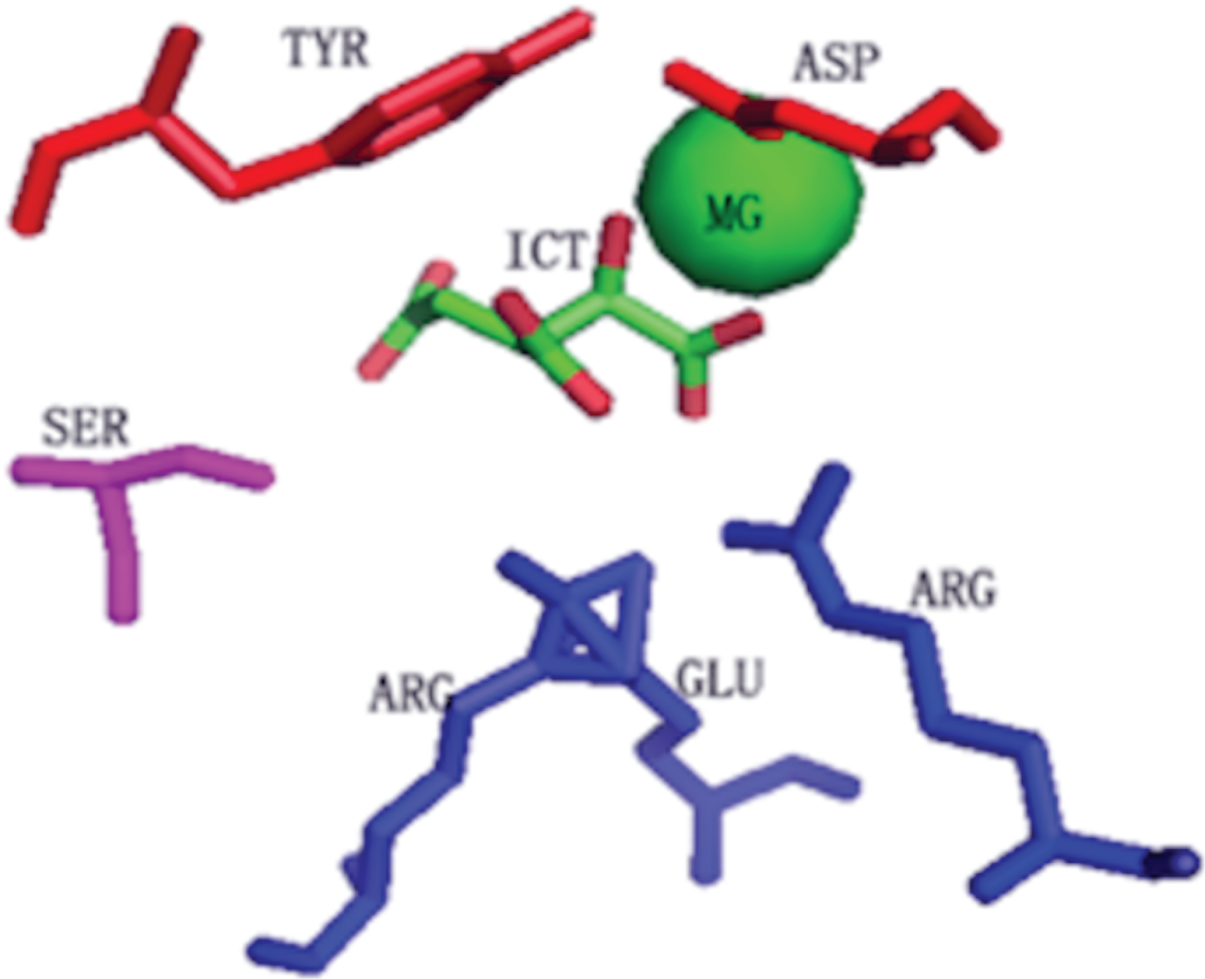
The 3D binding motif extracted by LioME for ICT under SR = 0.5. ICT and the conserved residues surrounded are rendered as sticks by PyMOL. The residues rendered in blue, red and fuchsia are supposed to interact with C1‐carboxyl and C2‐carboxyl, C2‐hydroxyl, and C5‐carboxyl, respectively. Please refer to the main text for details.

Figure 4 shows the motif when SR = 0.5, which displays the details of protein–ICT interactions. Two basic residues (ARG, rendered in blue) located in the vicinity of C1‐carboxyl (C1‐O1‐O2) and C6‐carboxyl (C6‐O5‐O6) of ICT, form a salt bridge with the two carboxyls. Two residues (TYR and ASP, rendered in red) and a MG ion (rendered in green) situated near the C2‐hydroxyl (C2‐O7). The hydroxyl of ASP may form covalent bond with MG ion together with the C2‐hydroxyl of ICT.

**Figure 5:**
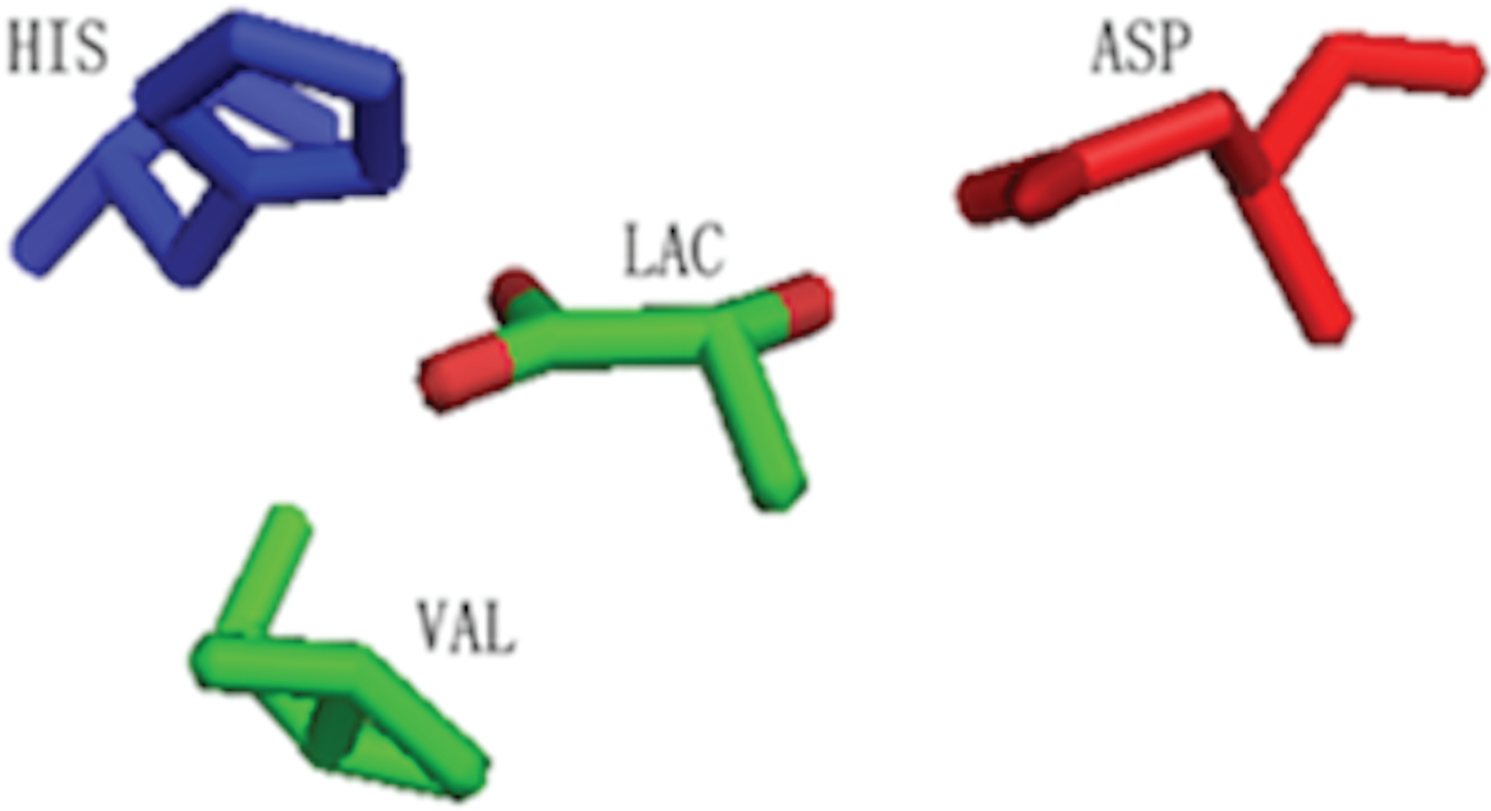
The 3D binding motifs for LAC extracted by LioME when SR = 0.5. LAC and the conserved residues surrounded are rendered as sticks by PyMOL.

TYR is expected to stabilize the local electric charge and provide hydrophobic interaction with the carbon skeleton. As for the polar residue (SER, rendered in fuchsia), it lies close to the C5‐carboxyl (C5‐O3‐O4) and a hydrogen bond is supposed to be formed.

The identified residue–ICT interactions present in the motif is consistent with the binding mode proposed by Mesecar *et al*. In their study, the authors indicated that the three attachments occur between ICT and its binding pockets (i.e., interactions with the three carboxyls) with the locality of the fourth group determining its stereospecificity (i.e., the interaction with hydroxyl) 23. We believe that the motif extracted for ICT is essential in the binding process.

### Motif extraction for ligands of small size

In the examples of ATP and ICT, we obtain conserved 3D binding motifs composing of submotifs that target specific functional groups of the ligands. For instance, a hydrophobic and A‐loop submotifs for the adenine base and a P‐loop submotif for the phosphate group are discovered in the case of ATP. As for ICT, hydrophobic submotifs targeting three carboxyls and one hydroxyl are identified, respectively. To further confirm the detection resolution of our method for functional groups, we try to extract binding motifs for small‐sized ligands with relatively less functional groups. LAC, with only one carboxyl and one hydroxyl, is selected as the model molecule. Figure 5 shows the binding motif under SR = 0.5.

As can be seen from the figure, three residues make up the binding motif with a polar residue (ASP) interacting with hydroxyl, a basic residue (HIS) interacting with carboxyl, and a hydrphobic residue (VAL) interacting with the carbon skeleton. It is obvious that patterns targeting for specific functional groups do exist as we have expected.

## Discussion

Many methods for ligand‐binding motif detection have been developed. Despite their efficiency for pocket comparison, classification, and prediction, these methods are not specifically designed to investigate protein–ligand recognition mechanisms. And many of these methods utilize the information of protein pockets only by an explicitly or implicitly comparative analysis of protein pockets. Here, we propose a method for protein–ligand‐binding motif discovery that combines information from the proteins and the ligands and provides details on protein–ligand interactions. The extracted motifs composed of residues of the proteins conserved in terms of amino acid types as well as relative positions to the ligand. From this information, we can easily obtain the knowledge about conserved protein–ligand interactions. For example, in the binding motif for ATP, we identify conserved interactions including C‐H–π interactions, π–π interactions, and hydrogen bonds regarding different functional parts of ATP that are proved to play different roles in the binding process 15, 16, 17, 18. In the case of ICT, conserved residues interacting with four main functional groups are revealed in the binding motif, which is consistent with the ‘four‐location’ mode proposed by Mesecar and Koshland 23. In addition, we extracted binding motif for LAC, a small‐sized ligand with only one carboxyl and one hydroxyl, the LAC‐binding motif also presents conserved interactions toward functional groups of LAC. This information could provide useful guidance to rational engineering and design of proteins targeting specific ligands.

A generally accepted point of view is that pockets with similar 3D structures could bind similar ligands. However, some studies also pointed out that protein–ligand recognition do not follow a one‐to‐one pattern but a multi‐to‐multi way 24, 25. That is, one ligand may display various binding modes when it binds to diverse receptors. Therefore, it is reasonable that different binding motifs/submotifs may exist for one specific ligand. Traditional motif extraction methods can only identify a ligand‐binding motif for a specific ligand. In contrast, different submotifs might be identified by our method for a specific ligand with different values of SR. The conservation of these motifs can be evaluated by their corresponding SR values. These clues shed light on a better understanding of the protein–ligand‐binding process. For instance, the hydrophobic submotif for ATP is the most conserved with highest SR values among all the residues in the binding motif. It is widely believed that hydrophobic interactions are a very important driving force in molecular recognition 26. So it is rational that the hydrophobic interactions are the most essential driving force in the binding of ATP. As we can see in Table 3, the same situation is observed in the binding of LAC, whose most conserved binding motif is also hydrophobic. As for ICT, a salt bridge with C1‐carboxyl and a hydrogen bond with C6‐carboxyl are the most conserved, indicating that the binding might be driven through two kinds of interactions from the ‘head’ and the ‘tail’ of the ligand, respectively. The metal ion interacting with hydroxyl is also much conserved since its interaction with C2‐hydroxyl of ICT determines the stereospecificity.

**Table 3:** The 3D binding motifs extracted by LibME for ICT and LAC under SR of 0.5, 0.6, 0.7, and 0.8, the conserved residues surrounded are rendered as sticks

Ligand	SR = 0.5	SR = 0.6	SR = 0.7	SR = 0.8
ICT	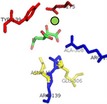	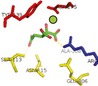	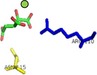	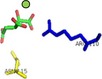
LAC	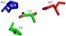	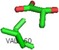	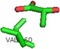	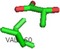

Another advantage of LibME is reflected in its applicability, which is widely broadened by the following two facts: First, we conquer the shortness of methods based on ligand‐induced superimposing of proteins. By incorporating relative positions to ligands, LibME is expected to extract binding motifs targeting flexible part of a ligand. Second, enough number of protein–ligand complexes are often required to extract precise binding motifs for a specific ligand. Despite data deficiency, it is possible for LibME to obtain useful information by utilizing data of similar ligands. As described above, in the cases of ATP, GTP, and AMP, we got identical submotifs targeting specific functional groups. One can alternatively seek a ligand with abundant data while containing similar functional groups as the target ligand when data for the target ligand is lacking. The identified submotifs may also provide useful information for the binding of the target ligand. In addition, this approach is also applicable for some large ligands with a high level of flexibility whose binding motifs extracted through our method directly are not accurate.

In summary, our method is not efficient for tasks like ligand prediction, since it is designed to assist the study of protein–ligand recognition mechanisms by identifying conserved protein–ligand interactions together with visual investigation. We believe that LibME can be a beneficial supplement to the existing motif extraction methods. Besides, LibME is applicable to any kind of ligand in theory, showing its potential to be a universal computational tool for extracting biologically meaningful 3D motifs.

## Conclusions

We present LibME, a method for extracting 3D protein–ligand‐binding motifs by integrating information from both the protein and the ligand. LibME extracts from a set of proteins binding the same ligand the residues situated around the target ligand that are conserved in terms of amino acid type as well as spatial positions. It can be applied to binding motif discovery and provides abundant information about protein–ligand interactions. The analysis of motifs generated by LibME will no doubt permit better understanding of protein–ligand recognition process, which in turn, will guide our rational design of proteins and drugs.

Our future work is to explore general principles that govern protein–ligand recognition through motif‐based large‐scale analysis. It is expected that more knowledge about protein–ligand interactions can be obtained with information provided by motifs for a large number of ligands extracted by our method.

## Author contributions

WH, ZL, MT, and LN conceived and designed the project, WH acquired the data, WH and ZL analyzed and interpreted the data, WH, ZL, MT, and LN wrote the paper.

## Supporting information


**Table S1.** Datasets of different ligand‐binding proteins.
