# Supplementary material for: LibME—automatic extraction of 3D ligand‐binding motifs for mechanistic analysis of protein–ligand recognition
Source: FEBS Open Bio. 2016 Nov 30;6(12):1331–40. doi: 10.1002/2211-5463.12150 (PMC5324770; doi:10.1002/2211-5463.12150)
Supplement: Supplementary file 1 — Table S1. Datasets of different ligand‐binding proteins. [file FEB4-6-1331-s001.docx]

**TS1: Datasets of different ligand-binding proteins.**

| PDB chains binding ATP (50 chains) for training:  1A0I_A, 1H3E_A, 1JI0_A, 1QHH_A, 1TF7_A, 1UA2_A, 1Z7E_A, 2CG9_A, 2HVY_A, 2J9L_A, 2JJX_A, 2PBZ_A, 2R6G_A, 2W00_A, 2WPD_A, 2YJE_A, 3A8T_A, 3AMT_A, 3BJU_A, 3D2E_A, 3DWL_A, 3E1Y_A, 3EA0_A, 3EPS_A, 3FKQ_A, 3FVQ_A, 3H1Q_A, 3HGM_A, 3INN_A, 3J2T_A, 3K5H_A, 3LKK_A, 3LY6_A, 3MN7_A, 3QB0_A, 3S3T_A, 3T54_A, 3VNQ_A, 3VX4_A, 3WBZ_A, 3ZC7_A, 3ZCN_A, 3ZIA_A, 4AI6_A, 4B1Z_A, 4BJR_A, 4DIN_A, 4DXL_A, 4EJ7_A, 4GXQ_A  PDB chains binding ATP (50 chains) for testing:  2Q0D_A, 2Q7G_A, 2QK4_A, 2R7L_A, 2VHQ_A, 2W00_A, 2X0Q_A, 2Y27_A, 2YCH_A, 2Z1U_A, 2ZSF_A, 3A8T_A, 3AM1_A, 3AQN_A, 3ATT_A, 3BG5_A, 3C4W_A, 3C9R_A, 3CQD_A, 3DKC_A, 3E1Y_A, 3EHG_A, 3ETH_A, 3FKQ_A, 3G59_A, 3GBU_A, 3H39_A, 3H8V_A, 3HMN_A, 3IBQ_A, 3IKH_A, 3INN_A, 3J1F_A, 3K5H_A, 3LFZ_A, 3LKK_A, 3LMI_A, 3MEY_A, 3NH9_A, 3Q60_A, 3QB0_A, 3QUN_A, 3QXC_A, 3R5F_A, 3RGL_A, 3RRF_A, 3SEZ_A, 3SUC_A, 3TUT_A, 4AUI_A  PDB chains binding AMP (48 chains):  1CJA_A, 1H3D_A, 1LTK_A, 1RY2_A, 2C5S_A, 2DCL_A, 2GM3_A, 2J91_A, 2Q2T_A, 2RIF_A, 2VSO_A, 2VZE_A, 3DDJ_A, 3EPS_A, 3ERR_A, 3FHM_A, 3FNA_A, 3FWZ_A, 3HF7_A, 3IUY_A, 3KD6_A, 3KGD_A, 3KH5_A, 3L2P_A, 3L31_A, 3L4B_A, 3LHH_A, 3LOQ_A, 3M84_A, 3N8H_A, 3NGN_A, 3NRN_A, 3NYQ_A, 3NZT_A, 3PCO_A, 3SZQ_A, 3UQ6_A, 3W1B_A, 4DG8_A, 4EEI_A, 4EQ5_A, 4FRY_A, 4HG0_A, 4HV4_A, 4IMY_A, 4LQY_A, 4M0K_A, 4NDF_A  PDB chains binding GTP (19 chains):  1C4K_A, 1CKM_A, 1UPT_A, 1VST_A, 2BKU_A, 2BTO_A, 2C5L_A, 2J59_A, 2WKP_A, 2X19_A, 3DOE_A, 3NWY_A, 3RSB_A, 3VHX_A, 4ARZ_A, 4DU6_A, 4F61_A, 4I4T_A, 4KGK_A  PDB chains binding ICT (10 chains):  1C97_A, 1ITW_A, 1LWD_A, 1T0L_A, 1P8F_A, 1W7F_A, 1W8G_A, 1XG4_A, 1XKD_A, 2UXR_A  PDB chains binding LAC (10 chains):  1C0K_A, 1K87_A, 1QH9_A, 1W3Q_A, 2FLT_A, 2FN7_A, 2IMP_A, 2NLI_A, 2P1E_A, 2ZZV_A |
| --- |

Note: PDB chains are described by their PDB codes followed by chain numbers
